# How to Teach Generative Artificial Intelligence in Undergraduate Medical Education

**DOI:** 10.1111/tct.70420

**Published:** 2026-04-09

**Authors:** Eva Feigerlova

**Affiliations:** ^1^ Centre de Référence des Maladies Héréditaires du Métabolisme, Centre Hospitalier Universitaire de Nancy Nancy France; ^2^ Faculté de Médecine, Maïeutique et Métiers de la Santé Centre Universitaire d'Enseignement Par Simulation – CUESim Vandœuvre‐lès‐Nancy France; ^3^ INSERM, UMR_S 1256 ‐ NGERE, Université de Lorraine Vandœuvre‐lès‐Nancy France

**Keywords:** artificial intelligence, learning modules, undergraduate medical curriculum

## Abstract

Generative artificial intelligence (AI) refers to computational systems capable of analysing data, recognising patterns and generating outputs that may support decisions. In healthcare, AI has the potential to improve diagnostic accuracy and provide clinical decision support. As AI becomes ubiquitous in clinical workflows, clinical teachers must be prepared not only to use AI tools but also to interpret, appraise and oversee their outputs safely and effectively. However, evidence indicates that medical curricula have not kept pace with technological adoption; structured AI education remains sparse or inconsistent across institutions. To address this gap, educators must define what medical students should learn about AI and how to teach it. Whereas existing literature defines what learners should know about AI, our work provides a pragmatic framework for how they should learn to use it in practice. By integrating verification, critical appraisal and ethical reflection into everyday clinical teaching, our workflow offers a scalable and adaptable model for preparing future clinicians to engage safely and responsibly with generative AI.

## 1 Introduction

Recent reviews synthesising AI curriculum frameworks and educational programmes found that although interest in AI education is growing, formal curricula remain scarce [1, 2]. Medical students are already using generative AI tools, often informally, for studying, drafting notes and summarising information. Without formal teaching, students may over‐rely on AI outputs, fail to recognise bias or misunderstand uncertainty in probabilistic predictions [3, 4]. Only a few teaching programmes reported explicit instructional frameworks, and most lacked theoretically grounded pedagogies or detailed learning outcomes [1, 2]. Students are rarely taught to cross‐check outputs against clinical guidelines or primary literature, nor to assess the reliability and uncertainty of model‐generated responses [3, 4]. Curricula also inadequately address issues of disclosure and accountability, leaving students without clear guidance on when and how to report AI use in clinical documentation or academic work [3, 5]. Despite widespread informal use of LLMs by students, opportunities for supervised, hands‐on engagement remain scarce, contributing to a hidden curriculum in which learners adopt AI tools without formal instruction or feedback [6]. Importantly, most current approaches remain conceptual or didactic, with limited emphasis on how learners should actively use, interrogate and integrate generative AI within real clinical workflows [3, 5, 6, 7]. In addition, there are no clear guidelines for evaluating the quality of AI‐based educational tools [2].

Expert interviews in medical AI education highlight essential learning domains: understanding AI fundamentals; interpreting and critically reflecting on AI output; ethical concerns, including privacy and bias; and practical skills for applying AI in clinical contexts [8]. Tolentino et al. indicate the need for interdisciplinary work, including educators, clinicians and data scientists, to develop integrated AI curricula designed for undergraduate medical education rather than isolated elective courses [1]. Shimizu et al. [9] highlight several positive aspects of learning with generative AI, such as improved learning efficiency, data gathering and case‐based learning.

This document proposes tips for guiding curriculum development across medical training stages that integrate the foundational, practical, experimental and ethical dimensions of AI‐based education. It adds value beyond existing guidance, providing a concrete structure and implementation tools designed for clinical teachers.

## 2 Defining Core AI Competencies for Medical Students

To ensure curricular coherence, AI instruction should be anchored in a clearly articulated competency framework [10]. Core competencies include (i) basic understanding of AI concepts; (ii) familiarity with generative AI tools in different clinical specialties; (iii) skills to interpret performance metrics of AI models, identify bias and evaluate clinical relevance of generated data; (iv) understanding ethical concerns; and (v) ability to integrate AI outputs into clinical reasoning, communicate AI‐informed recommendations to patients and colleagues and collaborate with multidisciplinary teams. These competencies can be articulated as progressive milestones, from introductory understanding in preclinical years to integration and application during clinical rotations.

## 3 Curriculum Design and Integration

A longitudinal spiral curriculum integrated throughout the medical studies is more appropriate for AI‐based education than stand‐alone educational content [10]. A spiral design allows integration of educational concepts with increasing complexity, reinforcing knowledge over time, for instance:

- Introduction of foundational generative AI principles in preclinical years through lectures, online modules and interactive tutorials that demystify machine learning and AI's clinical relevance.
- Contextualisation of AI in specialty rotations in clinical years (e.g., using AI tools for image interpretation in radiology or clinical decision support during ward rounds).
- Optional advanced projects or research experiences where students analyse real AI tools or datasets or design AI‐enhanced clinical workflows.

**FIGURE 1:**
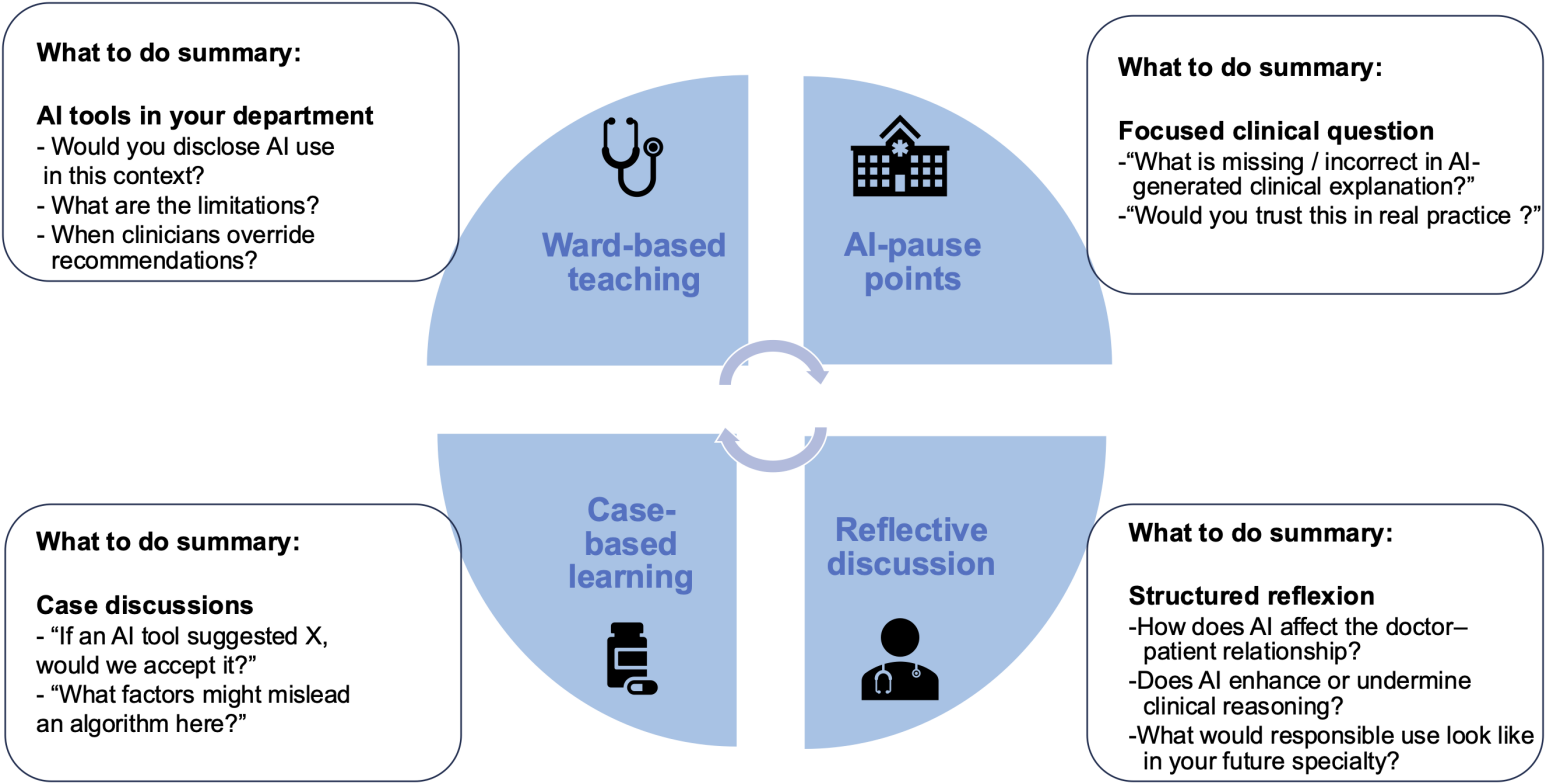
Practical strategies for clinical teachers.

This scaffolding strategy aligns with recommendations that AI curricula should be integrated into existing medical training [1]. The incorporation of AI topics into existing courses (e.g., ethics and evidence‐based medicine) rather than adding stand‐alone units mitigates a work overload. The objective for clinical teachers is not to transform their students into data scientists. Instead, clinical teachers should foster students' AI literacy and incorporate AI teaching into routine clinical supervision. Some examples are provided in Figure 1, and structured implementation protocols in Table S1. AI tools used in clinical practice can be discussed with the medical students to engage their critical thinking. For example, AI pause points (mirroring diagnostic pauses) introduce brief, repeatable moments of critical interrogation during ward rounds, whereas AI case‐based learning preserves the pedagogical strengths of case‐based learning by positioning AI as a comparator rather than a replacement for clinical reasoning. Structured reflective discussion sessions foster ethical awareness, addressing calls in the literature for deeper engagement with issues of accountability, disclosure and professional responsibility [3].

## 4 Instructional Methods and Teaching Strategies

Passive lectures are insufficient for complex topics such as AI‐based technologies. Among effective learning strategies that can be introduced are case‐based discussions, problem‐based learning and structured demonstration of generative AI tools under supervision by instructors. Uncertainty and bias can be explored through simulation‐based activities [4, 6, 11]. A key barrier to integrating generative AI into clinical teaching is time pressure. Short, structured interventions may be recommended as a feasible approach to embedding AI literacy into routine workflows (Table 1) [4, 6, 11].

**TABLE 1:** Short, structured interventions using generative AI.

	Quick guide for educators	
Example prompts (ready to use)	Providing structured prompts improves interaction quality and reduces ambiguity [11] Diagnostic reasoning: ‘What are the differential diagnoses for a 70‐year‐old with acute shortness of breath and chest pain?’ Management: ‘What is the initial management of suspected sepsis in an adult patient?’ Explanation: ‘Explain the causes of microcytic anaemia in a clinically relevant way’	
Verification checklist for students	Teaching explicit verification frameworks addresses known deficits in AI appraisal skills [6] **VERIFY** **V**alidated? → Does this match guidelines? **E**rrors? → Anything incorrect? **R**elevant? → Fits this patient? **I**ncomplete? → What is missing? **F**air? → Any bias? **Y**our decision → Accept/modify/reject	
Collect quick feedback	Iterative, feedback‐driven implementation is recommended for integrating AI into curricula [6] **Students:** Confidence in evaluating AI (Likert scale) Understanding of limitations (Likert scale) **Faculty:** Feasibility (Likert scale) Educational value (Likert scale)	
Increase complexity over time	Progressive learning aligns with competency‐based AI education models [11]	
	**Stage**	**Focus**
	Early	Error detection
	Intermediate	Bias, uncertainty
	Advanced	Ethics, accountability
Close the loop with learners	Reflection improves integration of AI into clinical reasoning [4] Ask: ‘How has your use of AI changed?’ ‘What safeguards do you now apply?’	

## 5 Blended Learning Approaches

Blended learning combines self‐paced online learning with in‐person interactive sessions [12]. Online modules cover AI basics (machine learning workflows, data types and model evaluation). In‐person expert‐led sessions, such as seminars and workshops, introduce students to generative AI tools and enable them to critique AI outputs. Sessions focused on problem‐based learning foster students' capacities to evaluate AI outputs in clinical scenarios, develop differential diagnoses and reflect on AI reliability and limitations.

## 6 Experiential Learning and Simulation

Practical exposure enhances comprehension through experience, conceptualisation and experimentation in a cyclical process [13]. Students interact with constraint‐based AI assistants or AI diagnostic tools under supervision, learn how to query systems, check outputs and integrate findings [10]. Active experimentation allows students to test and apply newly learned concepts. AI‐based tools can be used in different contexts: AI tutor (to increase knowledge), AI coach (to increase metacognition), AI mentor (to provide feedback), AI team member (to increase collaborative intelligence), AI student (to check understanding) or AI simulator (to assist with practice). Virtual patients or AI‐assisted simulators facilitate practice in clinical decision‐making augmented by AI recommendations. Table 2 illustrates the different pedagogical approaches using AI‐based tools as well as their educational advantages and disadvantages.

**TABLE 2:** Potential benefits and risks of different AI‐based pedagogical approaches.

Use of AI	Educational benefits	Educational risks
AI‐tutor	Personalised direct teaching is highly effective	The risk of confabulation
AI‐coach	Opportunities for reflection that promote learning	The supervisory style does not match the student's profile, with a risk of giving incorrect advice
AI‐mentor	Feedback promotes learning, even if all advices are not followed	The risk of confabulation
AI‐team member	Provides alternative perspectives to promote learning	The risk of confabulation and errors. Conflicts with team members
AI‐student	Teaching others is a method of learning	The risk of confabulation and errors
AI‐simulator	Practising and applying knowledge facilitates transfer	The risk of confabulation. Inappropriate fidelity. No single scenario will be effective for all students. Students may lose sight of the overall concept

## 7 Interprofessional Collaboration

AI education benefits from interdisciplinary teaching teams, combining expertise from clinicians, data scientists, ethicists and educators [1, 4]. Cross‐department collaboration fosters deeper understanding and bridges technical and clinical perspectives. It is necessary to systematically:

- Analyse the AI‐generated results to determine whether they correspond to educational objectives.
- Verify the accuracy and relevance of AI results against credible sources.
- Triangulate results between multiple AI tools.
- Recognise ethical risks such as AI‐generated misinformation and biased content and legal and ethical issues [2].

## 8 Assessment Strategies

Assessment should align with competencies and move beyond traditional testing:

- Performance tasks: Students interpret AI model outputs, critique study designs of AI research or evaluate case studies where AI tools influence clinical decisions. AI is prone to errors, hallucinations and biases, which must be checked [2, 14]. The risks associated with the use of AI are summarised in Table 3.
- Objective structured clinical exams (OSCEs): Scenarios include AI‐generated recommendations that students must assess and communicate appropriately (Table S1). Students learn how to create and test scenarios using prompts. One such example is illustrated in Table 4.
- Project portfolios: Capstone projects where students propose evaluations of existing AI tools or develop plans to integrate AI into clinical practice.

**TABLE 3:** Risks associated with the use of AI [2, 14].

Risk of confabulations (‘hallucinations’)	LLMs tend to produce incorrect but plausible facts. These are difficult to detect The error rate for this type varies from one LLM to another (in general, GPT‐4 and Bing have the lowest error rates). They are most common when asking for citations. The risk is highest with AI tutors and lowest with AI students
Risk of bias	AI is trained on a large amount of textual data and then receives additional training from humans. Both processes can introduce bias into the text, including gender and racial prejudice
Educational risk	AI can be very persuasive and have a very clear‐cut ‘viewpoint’ on the facts. There remains a significant risk that students will use AI as a crutch, thereby undermining learning
Risk related to personal data	The data implemented into AI‐based tools can be used for future AI training. The current state of privacy protection remains unclear for many models, and the legal implications are often uncertain as well

**TABLE 4:** Teaching modality AI‐simulator (example of prompt).

Prompt component	Description
Role/identity	You will play the role of the standardised patient, Mr Bilek, aged 35, who is consulting for a fever. I will play the role of the medical student replacing your general practitioner
Context	We are in a general practitioner's office, sitting opposite each other. This is the first time you have seen the student. You have a fever of 38.5°C, muscle and joint pain and have been sweating for 4 days. For the past 2 days, you have also had chest pain, which worsens when lying down and improves when sitting upright and leaning forward. You have been taking 1 g of paracetamol 3 times a day, but the fever keeps coming back. The pain has not changed
Invitation/question	Your objective is to allow medical students to practise interviewing an adult patient with a fever, focusing on questions that explore the symptoms. Do not play both roles. Do not ask questions spontaneously. Wait for the student to ask you the question. Wait for the student to answer before continuing the conversation. After four interactions, ask the student what the likely diagnosis is. Then conclude by telling the student how he/she performed as a doctor and what he/she can improve
Output format	Display the conversation with the list of questions and answers
Examples	Align with local/national learning outcomes frameworks

Assessment rubrics should also measure critical thinking, ethical reasoning and clinical integration skills.

## 9 Ethical, Legal and Professional Considerations

AI‐based tools raise complex ethical issues. Physicians must understand algorithmic bias, transparency, data privacy, informed consent regarding AI use and accountability for AI‐assisted decisions [2]. Teaching ethics should involve case discussions, regulatory frameworks and exploration of real‐world controversies. A scoping review on teaching AI ethics underscores this need while recommending interactive teaching modalities for ethical AI education [15].

## 10 Challenges and Implementation Considerations

Many educators lack expertise in AI; faculty development programmes and interprofessional teaching teams are crucial. Several factors must be taken into consideration, such as the relevance of the model used or potentially abnormal conclusions drawn by algorithms. Partnerships with schools of data science, engineering and informatics can leverage shared knowledge. Building flexible content that emphasises foundational principles and critical appraisal prepares students to adapt to new tools as they emerge [10].

## 11 Concluding Remarks

Teaching AI to medical students is essential for preparing future physicians to safely and effectively engage with AI‐augmented healthcare. The goal is to cultivate AI‐literate clinicians who can understand the capabilities and limitations of AI, use AI tools safely and responsibly, anticipate and mitigate bias or harm and contribute to innovation in multidisciplinary teams. Existing literature consistently highlights the need for AI literacy, including understanding model capabilities, limitations and ethical implications [3, 6]. Our proposed strategies directly respond to these gaps by operationalising key competencies identified in the literature, particularly verification, critical appraisal, responsible use and automation bias in LLM‐assisted decision‐making [4]. A key distinction of our contribution lies in its practical, educator‐facing design. Rather than proposing high‐level competencies alone, we translate these into structured, time‐bound teaching protocols that can be readily embedded into routine clinical teaching without substantial curricular redesign.

## Author Contributions

**Eva Feigerlova:** conceptualization, validation, methodology, writing – original draft, writing – review and editing, resources, visualization, investigation, software.

## Funding

The author has nothing to report.

## Conflicts of Interest

The author declares no conflicts of interest.

## Supporting information


**Table S1:** Examples of structured implementation protocols: ward‐based teaching, AI‐pause points, case‐based learning and reflective discussions.

## Data Availability

Data sharing not applicable to this article as no datasets were generated or analysed during the current study.
