# Supplementary material for: How to Teach Generative Artificial Intelligence in Undergraduate Medical Education
Source: Clin Teach. 2026 Apr 9;23(3):e70420. doi: 10.1111/tct.70420 (PMC13063796; doi:10.1111/tct.70420)
Supplement: Supplementary file 1 — Table S1: Examples of structured implementation protocols: ward‐based teaching, AI‐pause points, case‐based learning and reflective discussions. [file TCT-23-e70420-s001.docx]

**Supplementary Table 1.** Examples of structured implementation protocols

| **Ward-based teaching** | **Implementation protocol** |
| --- | --- |
| Aim | To develop undergraduate medical students’ ability to **safely, critically, and effectively use generative AI in clinical contexts,** with emphasis on: verification of AI outputs, clinical reasoning integration, ethical use, disclosure, and accountability. |
| Preparation | 1) Faculty preparation  - Identify **appropriate clinical cases** - Select an approved AI tool (e.g., ChatGPT) - Prepare **reference materials** (guidelines, textbooks) for verification  2) Safety and privacy guidance Before starting, explicitly instruct students:  **- Do not input identifiable patient data**   - - Use only anonymized case summaries /clinical descriptions - - AI tools are **not clinical decision-makers** - - All outputs must be **verified independently**  3) Learner briefing (5–10 min) Session objectives: **Generate → Verify → Decide → Disclose**   - Use AI to support clinical reasoning - Critically appraise AI outputs - Decide whether to **accept, modify, or reject** outputs - Justify decisions using clinical evidence |
| Steps | **Step 1: Clinical Case + Independent Reasoning (10–15 min) without IA**  - Present a real or simulated patient case - Students: take history / review summary; formulate differential diagnoses / initial management plan - Purpose: preserve baseline clinical reasoning  **Step 2: AI Interaction + Output Generation (10–15 min)** Students (in pairs/small groups):   - Input a **structured, anonymized prompt** into the AI tool Example: “Provide differential diagnoses for a 65-year-old with chest pain, hypertension, and diabetes.” - Ask follow-up questions: diagnostic approach, management suggestions - Record AI outputs  **Step 3: Verification, Appraisal, and Decision (15–20 min)** Students critically evaluate AI output:   1. **Verification**: cross-check with guidelines, textbooks; identify inaccuracies or omissions 2. **Critical appraisal**: Is it complete? Biased? Overly confident? 3. **Decision**: Categorize: Accept - Modify - Reject 4. **Justification**: Provide clinical reasoning for decisions 5. **Disclosure discussion**: Would you disclose AI use in this context? Why/why not? |
| Common pitfalls and mitigations | \| Pitfall \| Mitigation \| \| --- \| --- \| \| Overreliance on AI outputs \| Enforce Step 1 (AI-free reasoning first) \| \| Failure to detect hallucinations \| Require **mandatory verification step** \| \| Input of identifiable patient data \| Pre-session privacy briefing + supervision \| \| Superficial engagement \| Use structured worksheet: accept/modify/reject \| \| AI seen as authoritative \| Faculty models skepticism and uncertainty \| \| Time overrun \| Use strict time-boxing per step \| |
| Evaluation | **Mini OSCE-Style Station Blueprint** **Station title:** AI-assisted clinical reasoning  Student reviews: a clinical case and an AI-generated response  **Student must:** Identify ≥2 inaccuracies  Decide accept/modify/reject  Justify decision  **Assessment domains:** Verification / Reasoning / Safety awareness **Faculty Observation Checklist**  Did student: - verify AI output?  - challenge incorrect information?  - maintain independent reasoning? |

| **AI-pause points** | **Implementation protocol** |
| --- | --- |
| Aim | To embed brief, structured **“AI pause points”** within routine clinical teaching to help undergraduate medical students:   - - critically evaluate AI-generated clinical information - - verify outputs against evidence - - reflect on safe, ethical, and accountable AI use - - integrate AI into (rather than replace) clinical reasoning |
| Preparation | 1) Faculty preparation  - Identify **natural pause points** in teaching (e.g. after history taking, before management decisions) - Prepare: - a short clinical question suitable for AI - - access to an approved AI tool (e.g., ChatGPT)   - - reference sources (guidelines, local protocols)  2) Safety and privacy guidance (mandatory) Before participation, students must be instructed:   - - Do **not enter identifiable patient data**   - - AI outputs are **assistive, not authoritative**   - - all outputs must be **verified before use in practice**  3) Learner briefing (5 minutes) Introduce the concept:  **AI Pause Point = a deliberate stop to question AI output**  Explain the **4-step mental model**: **Ask → Generate → Challenge → Decide**  Students should understand:   - - AI may be wrong, incomplete, or biased - - their role is to **interrogate**, not accept |
| Steps | Each pause point takes **~5–10 minutes** and can be repeated during a session.  **Step 1: Clinical Question + Prediction (2–3 min)** AI not used yet  At a natural pause in teaching:   - Faculty asks a focused question: “What are the likely causes of this patient’s anemia?” - Students: - give their own answers first   - - commit to a position   Purpose: anchor independent reasoning **Step 2: AI Generation (2–3 min)**  - A student inputs a **structured, anonymized prompt** into the AI tool - AI generates differential diagnosis / explanation / management - Output is shared with the group  **Step 3: Challenge and Decision (3–5 min)** Students actively critique:  **Challenge**: What is missing?   - - What seems incorrect or overconfident?  1. **Verify**- Compare with: guidelines / prior knowledge 2. **Decide**    - Categorize: Accept - Modify - Reject 3. **Reflect:** Would you trust this in real practice?    - Would you disclose using AI here? |
| Common pitfalls and mitigations | \| Pitfall \| Mitigation \| \| --- \| --- \| \| Students defer to AI authority \| Require **pre-AI answer (Step 1)** \| \| Superficial critique \| Use “accept/modify/reject” framework \| \| Time pressure on ward rounds \| Limit pause points to 1–2 per session \| \| Patient data entered into AI \| Reinforce privacy rules before each session \| \| AI seen as always helpful \| Prompt discussion of **when not to use AI** \| \| Passive learners \| Assign rotating roles: prompter, verifier, challenger \| |
| Evaluation | **Mini OSCE-Style Station Blueprint** **Station title:** AI-Pause Point Evaluation  Student is given: a clinical case and an AI-generated response  **Student must:** Identify ≥2 issues (error, omission, bias)  Decide accept/modify/reject  Justify reasoning  **Assessment domains:** Verification / Critical appraisal / Safety awareness **Faculty Observation Checklist**  Did student: challenge AI outputs?  verify information?  maintain independent reasoning?  demonstrate ethical awareness? |

| **Case-based learning** | **Implementation protocol** |
| --- | --- |
| Aim | To use case-based learning augmented by generative AI to develop students’ ability to:   - - integrate AI into **clinical reasoning** - **- verify and critically appraise** AI-generated outputs - - apply **safe, ethical, and accountable AI use** in clinical contexts |
| Preparation | 1) Faculty preparation  - Select **authentic or simulated clinical cases** aligned with curriculum objectives - Prepare: - key learning points   - - reference materials (guidelines, textbooks)   - - structured prompts for AI interaction - Ensure access to an approved AI tool (e.g. ChatGPT)  2) Safety and privacy guidance (mandatory) Before starting: Students must **not input identifiable patient data**   - Reinforce: - AI is a **support tool, not a decision-maker**   - - all outputs must be **verified independently**  3) Learner briefing (5–10 minutes) Explain session structure and expectations:  Introduce the framework: **Reason → Generate → Evaluate → Apply**  Clarify that students will: - first reason independently   - - then compare with AI - - critically evaluate differences - - decide how to use AI safely |
| Steps | **Total time: ~45–60 minutes** **Step 1: Case Analysis and Independent Reasoning (15–20 min) without AI**  - Present a clinical case (progressively if desired) - Students (in small groups): - identify key problems   - - generate: differential diagnoses     - investigation plan     - management strategy   Purpose: preserve clinical reasoning, establish baseline thinking **Step 2: AI-Augmented Exploration (10–15 min)** Students use AI to explore the same case:   - Input structured prompt (anonymized), e.g.:“What are the differential diagnoses and management options for [case summary]?” - Ask follow-up questions: “What are the red flags?”   - “What is the recommended investigation pathway?” - Document AI outputs  **Step 3: Critical Appraisal and Clinical Integration (15–20 min)** Students compare their reasoning with AI output: 1. **Verification**  - Cross-check with: guidelines /textbooks - Identify: errors, omissions  2. **Critical appraisal**  - Assess: completeness, bias, appropriateness  3. **Integration**  - Decide: - what to adopt from AI   - - what to reject - Refine original clinical plan  4. **Reflection and disclosure discussion**  - Would AI use be appropriate in real practice? - Would it need to be disclosed? |
| Common pitfalls and mitigations | \| Pitfall \| Mitigation \| \| --- \| --- \| \| Students rely on AI too early \| Enforce **AI-free Step 1** \| \| Superficial comparison with AI \| Require structured “compare and justify” exercise \| \| AI output accepted uncritically \| Use **accept/modify/reject framework** \| \| Lack of engagement \| Assign roles (leader, prompter, verifier, presenter) \| \| Time constraints \| Limit number of AI queries \| \| Privacy breaches \| Reinforce anonymization rules \| |
| Evaluation | **Mini OSCE-Style Station Blueprint** **Station title:** AI-supported case evaluation  Student reviews: clinical case and AI-generated response  **Student must:** Identify ≥2 issues (error, omission, bias)  Decide accept/modify/reject  Justify decisions  **Assessment domains:** Reasoning / Verification / Safety awareness **Faculty Observation Checklist**  Observers assess: - degree of independent reasoning   - - ability to critique AI outputs - - safe integration into decision-making |

| **Reflective discussions** | **Implementation protocol** |
| --- | --- |
| Aim | To develop students’ ability to **critically reflect on the use of generative AI in clinical practice**, with emphasis on:   - - identifying strengths and limitations of AI outputs - - understanding risks (e.g., hallucinations, bias, overreliance) - - applying **ethical principles, disclosure, and accountability** - - integrating AI into clinical reasoning **without loss of professional judgment** |
| Preparation | 1) Faculty preparation  - Select **trigger materials**, such as: - - a clinical case with AI-generated response   - examples of flawed or biased AI outputs   - - - scenarios involving ethical dilemmas (e.g., undocumented AI use) - Prepare: - guiding questions   - - relevant clinical guidelines or references - Ensure access to an approved AI tool (e.g., ChatGPT)  2) Safety and privacy guidance (mandatory)  - No identifiable patient data to be entered into AI tools   - AI outputs must be **verified independently**   - AI is **assistive, not authoritative**   - Responsibility remains with the clinician  3) Learner briefing (5–10 minutes) Introduce session purpose: “This session is about how you think about AI, not just how you use it.”  Present reflective framework: **Experience → Analyze → Challenge → Apply**  Clarify expectations: - active participation   - - openness to uncertainty - - critical, not passive, engagement |
| Steps | **Total time: ~45–60 minutes** **Step 1: Trigger and Individual Reflection (10–15 min)**  - Present: a clinical case + AI-generated output or a real scenario where AI influenced decision-making - Students individually reflect: What is useful?   - What is concerning?   - Would I trust this? Why/why not?  **Step 2: Group Discussion and Critical Exploration (20–25 min)** Facilitated small-group or whole-group discussion: Key prompts:  - **- Accuracy and reliability**: What errors or omissions are present? - **- Bias and safety**: Could this output disadvantage certain patients?   - What risks arise if used uncritically? - **- Cognitive impact**: Did AI influence your thinking?   - Would you have reasoned differently without it?   **Ethics and professionalism**: Should AI use be disclosed here?   - - Who is accountable if AI is wrong?  **Step 3: Synthesis and Application (10–15 min)** Students develop practical takeaways: When is AI helpful vs unsafe?   - What safeguards are needed? - How will I use AI differently in future?   Faculty summarizes key principles:   - - Verify before acting - - Maintain independent reasoning - - Be transparent when appropriate - - Accept responsibility for decisions |
| Common pitfalls and mitigations | \| Pitfall \| Mitigation \| \| --- \| --- \| \| Superficial discussion \| Use structured prompts (accuracy, bias, ethics) \| \| Students defer to AI authority \| Highlight known AI errors \| \| Discussion drifts off-topic \| Keep focus on clinical relevance \| \| Lack of engagement \| Use individual reflection before group discussion \| \| Ethical issues treated abstractly \| Use real-world clinical scenarios \| \| Dominance by few students \| Use small groups or round-robin responses \| |
| Evaluation | **Mini OSCE-Style Station Blueprint** **Station title:** AI-ethics and critical reflection  Student reviews a clinical case and AI-generated recommendation  **Student must:** Identify risks (e.g. bias, inaccuracy)  Discuss ethical considerations  Explain appropriate use  **Assessment domains:** Insight /Ethical reasoning/ Safety awareness **Faculty Observation Checklist**  Observers assess whether students:   - - engage critically with AI outputs - - recognize ethical and safety issues - - demonstrate reflective thinking - - connect discussion to clinical practice |
